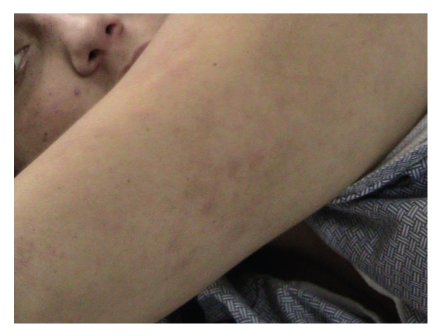# Adult Onset Still's Disease and Rocky Mountain Spotted Fever

**DOI:** 10.1155/2010/621046

**Published:** 2010-08-09

**Authors:** Paul Persad, Rajendrakumar Patel, Niki Patel

**Affiliations:** Department of Internal Medicine, Mercer University School of Medicine, Macon, GA 31201-0001, USA

## Abstract

Adult Still's Disease was first described in 1971 by Bywaters in fourteen adult female patients who presented with symptoms indistinguishable from that of classic childhood Still's Disease (Bywaters, 1971). George Still in 1896 first recognized this triad of quotidian (daily) fevers, evanescent rash, and arthritis in children with what later became known as juvenile inflammatory arthritis (Still, 1990). Adult Onset Still's Disease (AOSD) is an inflammatory condition of unknown etiology characterized by an evanescent rash, quotidian fevers, and arthralgias. Numerous infectious agents have been associated with its presentation. This case is to our knowledge the first presentation of AOSD in the setting of Rocky Mountain Spotted Fever. Although numerous infectious agents have been suggested, the etiology of this disorder remains elusive. Nevertheless, infection may in fact play a role in triggering the onset of symptoms in those with this disorder. Our case presentation is, to our knowledge, the first case of Adult Onset Still's Disease associated with Rocky Mountain spotted fever (RMSF).

## 1. Case Report

A twenty-one-year old white female was admitted to our internal medicine service for arthralgias and myalgias of two weeks duration. For the past week, she noted daily fevers and almost simultaneous rash. Associated symptoms included sore throat, nausea, and vomiting. Her arthralgias involved her wrists, ankles, and knees bilaterally. Patient stated that she had been hiking in the North Georgia Mountains in March and removed a tick from her leg and her pubis approximately three days before her joint symptoms began. She was unaware of how long the tick had been on her body. She denied noticing any rashes at the site of tick removal. After about one week's time, she noted feeling feverish and that a rash had developed on her right thigh, arm, and torso (see figure).

Past medical and surgical history was negative. Patient admitted to occasional alcohol and tobacco abuse but denied illicit drugs. She denied history of sexually transmitted diseases other than Bacterial Vaginosis but admitted to sexual abuse by recent ex-boyfriend. Current medications included metronidazole prescribed by emergency room physician, Tylenol, and Benadryl. Patient stated that penicillin caused a “bad rash” as a child. Physical exam was as follows.

**Vital Signs**

Blood Pressure was 142/72, pulse was 81 beats per minute and regular, respiratory rate was 18 breaths per minute, and temperature was 102 degrees Fahrenheit.

**Figure 1:**
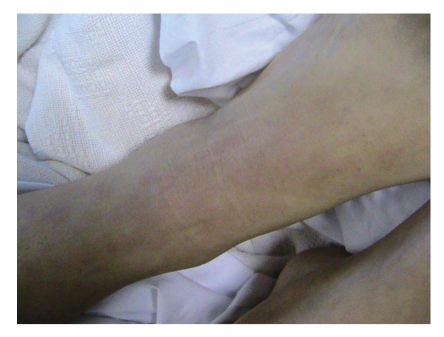


Oral exam revealed an erythematous pharynx without tonsillar exudate, mild conjunctival injection, mild bilateral cervical lymphadenopathy, and modestly tender abdomen with a mildly enlarged spleen. Elbows, shoulders, hips, and knee joints were tender to palpation and passive motion bilaterally. A salmon colored raised rash was noted on her trunk, arms, and legs (see Figures 1 and 2). Laboratory exam was as follows.

**WBC Count**

13.93 K cells/mm^3^ with a neutrophillic predominance (83%), Hemoglobin: 11.2 gm%, Mean corpuscular volume: 89.9 fL, platelets: 233 K/mm^3^, AST: 134 U/L, ALT: 156 U/L, LDH: 430 U/L, CRP: 21.1 mg/dL, ESR: 119 mm/hr, ANA and Rhematoid Factors were both negative. Blood cultures were negative. Rocky Mountain Spotted Fever Titers measured via Latex agglutination assay were positive at 1 : 16 on admission and increased to 1 : 64 on subsequent testing. HIV test was negative. Blood and urine cultures were negative. Serology for Mycoplasma and Chlamydia was negative. Vaginal and rectal swabs for gonorrhea were negative. Computerized Tomography of the Abdomen and Pelvis was significant for moderate splenomegaly and mesenteric adenitis.

**Hospital Course**

Patient was admitted to the staff medicine service and received broad spectrum antibiotics including doxycycline 100 mg by mouth twice daily to cover tick borne illnesses including RMSF. This resulted in improvement in her joint symptoms but not in her fever or rash. Her rising RMSF titers, elevated liver enzymes, and symptom improvement with doxycycline were presumptive evidence that RMSF was culprit. Our patient's rash, however, was not characteristic of RMSF and its fluctuation with her fever was also suspicious. Careful evaluation of the rash and fever spikes revealed the quotidian nature of her disease (see Figures 1 and 2).

We found that our patient's current symptoms were consistent with Yamaguchi criteria for Adult Onset Still's disease. We checked her ferritin level which came back markedly elevated at 9362 ng/mL. After discussion with our Infectious disease and rheumatologic consultants we stopped antibiotics on day 7 and started the patient on naproxen 500 mg twice daily. This resulted in prompt resolution of her fevers and rash within 24 hours. Of interest, the patient refused two subsequent doses and her symptoms recurred. After reiterating the inflammatory nature of her disease, the patient resumed her naproxen with another marked abatement of her symptoms.

We feel that our patient's symptoms were not due to RMSF, because the rash was salmon colored and worsened with the fever spikes. Furthermore, her prompt improvement of all symptoms with anti-inflammatory agents and not doxycycline speaks against an infectious etiology. RMSF may have just been the trigger to this inflammatory condition.

## 2. Discussion

Adult onset Still's Disease (AOSD) is rare systemic inflammatory disorder of unknown etiology and pathogenesis. Numerous infectious agents have been proposed as potential inciting factors. Viruses such as Epstein-Barr virus, Coxsachievirus, adenovirus, influenza A, human herpes 6, hepatitis B, hepatitis C, and parvovirus B19 have been implicated in small case series [3]. Bacterial triggers may include Chlamydia pneumonia, Mycoplasma pneumonia, Yersinia enterocolitica, and Borrelia burgdorferi [4, 5]. These associations suggest that this disease entity may be similar to reactive arthritis [6]. Our patient represents to our knowledge the first case of AOSD associated with Rocky Mountain Spotted Fever (RMSF). 

### 2.1. Clinical Manifestations

AOSD classically presents with high spiking fevers and systemic manifestations [7]. Characteristic rash, joint or muscle pains, lymphadenopathy, and abnormalities of liver function may be seen [8]. The fever generally exceeds 39 deg C and is quotidian or double-quotidian in nature, peaking in the late afternoon [9].

The typical rash is often described as evanescent, salmon to pink colored, maculopapular occurring on the extremities and trunk sparing the face. Histology is often nonspecific but may demonstrate dermal invasion by lymphocytes and histiocytes with perivascular inflammation [10].

Arthritis and arthralgias are found in the vast majority of patients (64–100%) [11]. Ankles, knees, and wrists are the most commonly involved joints but elbows, shoulders and hips have also been described [12]. Wrist involvement may include joint space narrowing and ankylosis within 3 years of disease onset in some untreated cases [13]. Radiographs are usually not helpful early on in the disease.

Hepatic involvement characterized by abnormal liver function tests can be seen in up to 75% of patients [14]. Cardiac complications may include pericardial effusions and myocarditis [15]. Lung involvement may be represented by pleural effusions, fibrosis, or rarely acute respiratory distress syndrome [16].

### 2.2. Diagnosis

AOSD is a clinical diagnosis. Various diagnostic criteria have been developed including the Yamaguchi [17], Cush [18], and Fautrel criteria [19]. Antinuclear antibody (ANA) and Rheumatoid factor (RF) are classically negative. Erythrocyte sedimentation rate (ESR) and C-reactive protein (CRP) are usually positive reflecting the inflammatory nature of this condition [11, 20]. A striking neutrophilic (>80%) leukocytosis is often seen on blood work [10]. Hematophagic syndrome may be heralded by the onset of pancytopenia and requires prompt immunosuppressive therapy [21]. Recently ferritin has been considered in diagnostic criteria reflecting its role in oxidative stress [19]. A serum ferritin threshold of greater than 1000 ng/mL has been used to suggest AOSD [22]. The glycosylated fraction of serum ferritin has also been suggested as a more specific marker of the inflammation in AOSD [22]. Low levels of this fraction represents disease activity but cannot be used to follow progression or response to treatment.

The differential diagnosis of AOSD is broad. Seronegative spondyloarthropathies, reactive arthritis, granulomatous disorders, familial Mediterranean fever, and various vasculidities may present similarly. Infections, malignancies, and other rheumatologic conditions must be considered and ruled out.

### 2.3. Treatment

NSAIDs, corticosteroids, and antirheumatic agents are the cornerstones of therapy [11]. Intially NSAIDs such as naproxen or indomethacin are tried. Between 50 and 75% of those with progressive or recurrent symptoms will require steroids [10]. Patients with NSAID refractory disease, pancytopenia, pericarditis, pleuritis, or elevated liver function tests are particularly at risk for progression without steroid treatment [23]. Novel agents such as anti-TNF drugs, interleukin blockade, and intravenous gammaglobulin (IVIG) have been tried in small studies with limited success [24].

### 2.4. Course and Prognosis

Three patterns of disease progression have been described [24].

1. Monocyclic involvement is characterized by systemic symptoms of fever, rash, organomegaly, and seriositis. Most patients will have one episode of disease and achieve remission within one year.
2. Polycyclic pattern involves multiple recurrences that may or may not affect joints. Complete remission is usually achieved between flares that may be separated by many years. Recurrences are often milder than first attack.
3. Chronic articular pattern targets the joints primarily with minimal to no systemic symptoms. Severe joint destruction is often the end result in untreated cases.

### 2.5. Follow-Up with Our Case

Our patient was discharged home on naproxen and with a follow-up appointment for our staff medicine resident and staff rheumatology clinic. She did not attend either. Two months later she presented again to the Emergency Department with fever, rash, joint pains, and bilateral pleural effusions. These were drained and patient responded well to a course of steroids. Unfortunately, her ferritin was not checked by the admitting hospitalist. Radiographs of her involved joints were not revealing of ankylosis. She was rescheduled for Rheumatology Clinic one week after discharge. She again has not shown up for any of her appointments.

## Figures and Tables

**Figure 2:**